# Supporting early academic family medicine careers with the clinician scholar enhanced-skills program

**Published:** 2019-11-28

**Authors:** Miriam Lacasse, Annie St-Pierre, Andréane Lalumière-Saindon, Marie-Hélène Dufour, Anik Giguère, Guy Béland

**Affiliations:** 1 Department of Family Medicine and Emergency Medicine, Faculté de médecine, Université Laval, Québec, Canada; 2 Department of Family and Community Medicine, University of Toronto, Ontario, Canada

## Abstract

**Context:**

The Clinician Scholar Program (CSP) is an enhanced-skills (R3) residency program to train clinician researchers/educators/leaders for academic family practice. This article intends to share Laval University’s CSP development and evaluation strategy, and provide recommendations for similar innovations in other disciplines/settings.

**Methods:**

This article uses Kern’s model to present the program development, and a program-oriented approach for program evaluation, carried from 2011 to 2017 using descriptive data. Questionnaires, reflexive texts and an Objective Structured Teaching Exam supported data collection.

**Results:**

Seven CSP graduates and 14 controls participated in the program evaluation. Residents were highly satisfied with the program, nevertheless they suggested to allow physicians to come back for training later in career. The CSP enriched knowledge, skills and attitudes about academic practice. CSP increased residents’ entrustment level about academic competencies. All graduates joined an academic practice within five years of program completion.

**Conclusion:**

Key recommendations to implement similar programs include academic medicine core training, project- based learning with learner-centered objectives, relevant and authentic learning and assessment, and multi-level program evaluation approach. Programs should consider concomitant graduate studies and opportunity to offer such training after a few years of clinical practice to meet other needs at a timely stage of career.

## Abstract (fr)

**Contexte:**

Le programme clinicien érudit (PCÉ) est un programme de résidence de compétences avancées (R3) destiné à former des cliniciens chercheurs / éducateurs / leaders en vue d’une pratique de médecine familiale universitaire. Cet article a pour but de faire connaître la stratégie de développement et d’évaluation du PCÉ de l’Université Laval et de formuler des recommandations pour des innovations similaires dans d’autres disciplines/contextes.

**Méthodes:**

Cet article utilise le modèle de Kern pour présenter le développement du programme et une approche d’évaluation orientée sur le programme, réalisée de 2011 à 2017 à l’aide de données descriptives. Des questionnaires, des textes réflexifs et un examen d’enseignement objectif structuré ont permis de recueillir des données.

**Résultats:**

Sept diplômés du PCÉ et 14 témoins ont participé à l'évaluation du programme. Les résidents étaient très satisfaits du programme, suggérant néanmoins de permettre une formation plus tard dans la carrière. Le PCÉ a enrichi les connaissances, les habiletés et les attitudes relatives à la pratique universitaire. Le PCÉ a augmenté le niveau de confiance des résidents en ce qui concerne les compétences académiques. Tous les diplômés se sont engagés dans une pratique universitaire dans les cinq années suivant leur graduation du programme.

**Conclusions:**

Les principales recommandations pour la mise en œuvre de programmes similaires incluent la réalisation d’un tronc commun en médecine universitaire, l’apprentissage par projet avec des objectifs centrés sur l'apprenant, des stratégies d’apprentissage et d’évaluation pertinentes et authentiques, et une approche d'évaluation de programme à plusieurs niveaux. Les programmes doivent envisager offrir des études supérieures en parallèle et la possibilité d’offrir cette formation après quelques années de pratique clinique afin de répondre à d’autres besoins à un stade opportun de leur carrière.

## Introduction

Since the beginning of 21^st^ century, academic family medicine has been facing various challenges, such as adjusting to new clinical demands in academic health centers, organizing and administering new initiatives in community-based education, developing and maintaining research capacity, and serving multiple missions (education, clinical care, and academic pursuits) in times of financial restraint.^1^ Training and recruiting academic physicians is a major challenge, particularly early on in their career.^2^ The literature describes many research, education, and leadership training programs for early-career clinicians.^3^^,^^4^ However, few target residents or focus on more than one of these academic missions.

Increasing capacity in many Canadian family medicine residency programs in recent years has resulted in the hiring of a pool of clinician teachers, acting mainly as role models without necessarily having other graduate degrees besides the medical diploma. As in United States,^5^^,^^6^ family medicine programs are facing the challenge of training a new wave of physicians with an interest in academia.

Since 2009, the College of Family Physicians of Canada (CFPC) has been encouraging medical schools to offer a Clinician Scholar Program (CSP) as an enhanced-skills (supplemental year) program. Currently, most CSPs offered in Canada are research- oriented and offered in English. Université Laval’s CSP is intended to train clinician researchers, educators and leaders in clinical and academic francophone settings, promoting scholarship^7^ in all three academic missions.

As proposed by Thompson,^5^ this article intends to share our CSP development and first five years’ program evaluation strategy and provide recommendations for similar academic fellowship in other settings.

## Methods

### Program development and overview

The CSP aims to train competent faculty who are aware of the importance of scholarship as a way to enhance their institution’s national and international outreach, as well as their own. Residents involved in this program mostly complete it as a full-time PGY3, but may also spread it over PGY2 and PGY3 during completion of their second year of family medicine residency training. The CSP curriculum starts with a core Academic Medicine rotation where all residents are trained around academic practice requirements, strategies, and its expected scholarship. An elective in clinical research, medical education or academic management/leadership then allows them to enhance their skills in a one of these academic missions, and clinical activities are spread over the rest of the year (Table 1), to provide residents with a clinical and academic schedule similar to the one they will manage in their future practices.

**Table 1:** Université Laval’s clinician scholar program curriculum

Track			Teaching activities	Duration	Description	Teaching strategies		
**Research**	**Education**	**Leadership/ Management**				**In-class learning**	**Practicum**	**Research/ Innovation project**
x	x	x	**Academic medicine rotation** NOTE: This rotation also welcomes up to 8 residents from other CFPC or RCPSC residency programs to help build a community of practice and optimize resources.	12 weeks block (first 3 months of training)	12-week long pillar for the development of a range of basic research, teaching and leadership skills. Inspired mostly from humanist and socio-constructivist learning theories, this rotation promotes learners’ intrinsic motivation and responsibility for learning through a project-based approach that promotes scholarship and experiential learning, fostering learning through the creation of a community of practice.	28 three-hour long workshops focusing on each step of a scholarly project as well as on some medical education and leadership/ management skills (estimated faculty time around 80 hours, since some workshops involve more than one faculty)	Three hours of clinical supervision accompanied by a role model (estimated faculty time: 3h)	Drafting of project proposal (estimated faculty time for project supervision: 3h)
x	x	x	**Family medicine rotation**	12 weeks, horizontal	Enables residents to maintain 120 half-days of clinical practice spread over the entire year of training.		Family medicine clinical activities spread across the duration of the program (2-3 half-days/week). (estimated faculty time: around 30 minutes per half-day for overhead supervision)	
x			**Clinical research elective**	28 weeks, horizontal	28-week long elective, involving: • graduate-level courses • longitudinal practicum, where they are also encouraged (with financial support) to visit other medical schools as “academic tourists” to broaden their understanding of academic medicine. • research/innovation project to be presented in a local or national conference by the end of their training	Two master’s degree courses in clinical epidemiology or other relevant disciplines (already available to epidemiology students in other programs, so no extra time commitment for faculty)		Clinical research project (estimated faculty time: 50h)
	x		**Medical education elective**	28 weeks, horizontal		Master’s degree-level courses (Medical Education – Principles and Practices - estimated faculty time: 54h) and faculty development workshops (already available to all clinical teachers, so no extra time commitment for faculty)	Undergraduate teaching Clinical supervision (clerks and residents) Faculty development and/or continuing professional development Metasupervision by senior clinical teachers (estimated faculty time: 50h)	Needs assessment, curriculum development, program evaluation, etc. (estimated faculty time: 50h)
		x	**Medical and Academic Leadership/ Management elective**	28 weeks, horizontal		Two master’s degree courses in administration (already available to students in administration, so no extra time commitment for faculty)		Development, implementation and/or evaluation of a leadership/ management project (estimated faculty time: 50h)
x	x	x	Clinical scholar program lunches	8 one-hour meetings	Monthly encounters to enhance the skills developed during the Academic Medicine rotation while providing opportunities for networking with faculty.	Monthly discussion meetings on academic medicine-related topics. Before each meeting, participants do the suggested readings. During the activity, discussions on the topic takes place with invited faculty members (estimated faculty time: 8h)		

CFPC: College of Family Physicians of Canada; RCPSC: Royal College of Physicians and Surgeons of Canada

**Figure 1:**
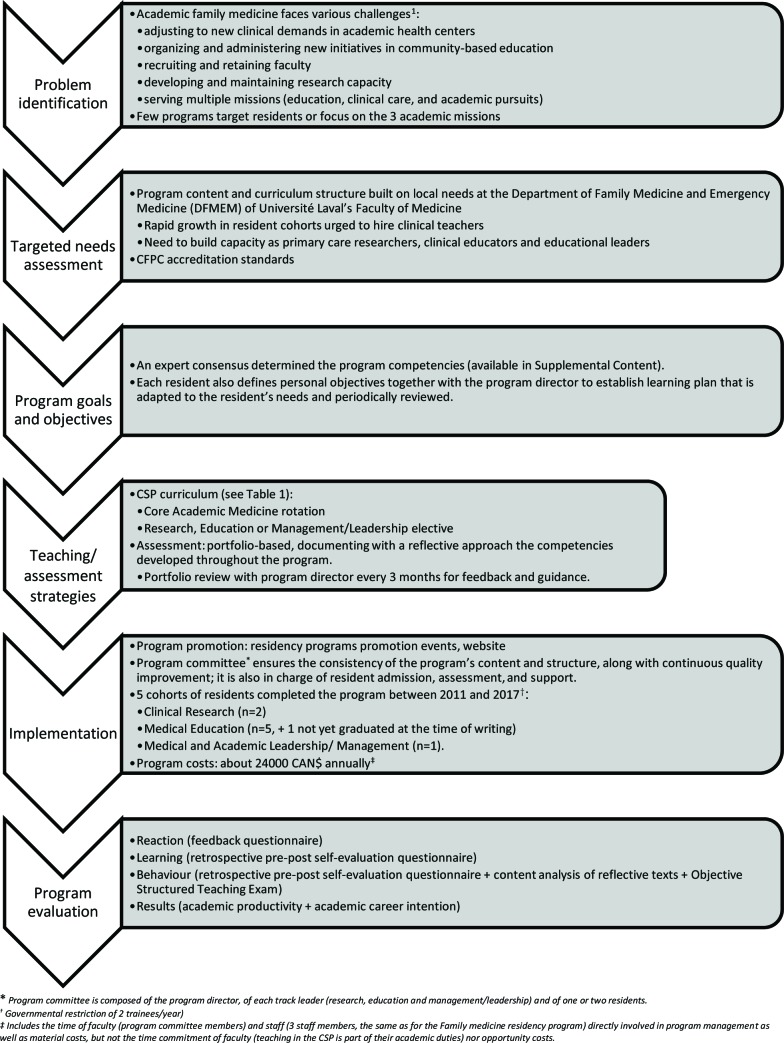
Program development (adapted from Kern’s steps of curriculum development^8^)

The Family medicine and emergency medicine department chair and the family medicine program director at Laval University initially approached the future CSP program director (who had recently completed the Academic Fellowship program at the Department of Family and Community Medicine, University of Toronto) to discuss local needs around training for academic practice. Needs assessment included discussions with local faculty, analysis of the CFPC standards for the new CSP programs, and a literature review. The program therefore built on similar existing programs.^2^^,^^5^^,^^6^ This article uses Kern’s steps to present the program development^8^ (Figure 1).

Presentation of the program to medical students at Laval University “Salon des programmes de residences”, program website (http://www.fmed.ulaval.ca/programmes-detudes/etudes-en-medecine/residences-etudes-medicales-postdoctorales/residence-en-clinicien-erudit/presentation/) and targeted solicitation by site directors facilitated resident recruitment.

Most faculty for the CSP had already completed graduate studies in research, medical education or management/leadership programs. The others were already involved as faculty development workshops facilitators. At the beginning of each academic year, personalized training around metasupervision is provided by the program direction to the preceptors in teaching units attended by CSP residents in the medical education track.

***Program evaluation*.** Program evaluation was carried out from 2011 to 2017 using descriptive data based on a program-oriented approach.^9^ Kirkpatrick’s classification of training outcomes^10^ structured the data collection. Although program evaluation activities do not fall within the scope of research ethics board review at our institution^1^, we respected voluntary participation (not mandatory for promotion), informed consent and confidentiality in data management and reporting results.

***Reaction***. We assessed reactions to the program activities using a feedback questionnaire at program completion, providing a satisfaction score (product of relevance (/5) x met expectations (/3)) and assessing general satisfaction and workload. Since the Medical and Academic Leadership/Management elective was particularly innovative, we analyzed the strengths and avenues for improvement of this rotation with a preceptor survey.

***Learning*.** We assessed learning using a retrospective pre-post self-evaluation questionnaire.^11^ Self-assessment scales regarding achievement of program evaluation objectives drew on Bloom’s (knowledge),^12^^,^^13^ Simpson’s (skills),^14^ and Krathwohl’s (attitudes)^15^ taxonomies. We also asked each resident to identify three take-home messages from the program.

***Behaviour***. We assessed the impact of the CSP on resident behaviour also using a retrospective pre-post self-evaluation questionnaire,^11^ as well as content analysis of reflective texts written by residents upon completing the program, applying a framework for analysis relating to the three academic missions.

Finally, we assessed the impact of the Medical Education track on residents’ teaching behaviours using Objective Structured Teaching Exams (OSTE) held at the outset and the end of the program, each learner being paired with another resident of equivalent level of training demonstrating a similar interest in education (control). The examination used Morrison et al.’s teaching scenarios,^16^ translated and adapted with permission. The assessment grid was adapted from the CFPC’s fundamental teaching activities framework.^17^

***Results*.** The “results” component of this program evaluation compares the scholarship of CSP graduates with other new clinical faculty who joined our department between 2011 and 2015 (control group), and their intention to practice in an academic setting five years of graduation. We assessed the factors influencing this intention with a social cognitive theory-based questionnaire.^18^

## Results

Five to seven CSP of the 7 graduates (71-100%) and 14 of the 20 new clinical faculty who joined our department between 2011 and 2015 (control group, 70% response rate) took part in the program evaluation (variable number of participants for each type of evaluation). One third of graduates and 20% of the control group held graduate degrees in addition to their medical degree.

### Reactions to the program

The general satisfaction with the program was high (4.7±0.5/5). The workload was appraised as demanding to very demanding (4.3±0.5/5). The most appreciated activity was the Academic Medicine intensive workshops (score 14.3/15, n=7). All graduates would recommend the program to colleagues interested in academic practice.

Program evaluation specific to the Medical and Academic Leadership/Management elective has highlighted the various levels of practicum exposure (*clinical*: local/regional/provincial; *academic*: teaching site/program/department/faculty level), as well as the resident project spinoffs for the teaching site. Suggested improvements included focusing on some activities and choosing a limited number of supervisors to enhance educational continuity, dispersion across activities and supervisors, better defining resident and supervisor roles and responsibilities, and allowing later enrollment into the program, i.e. after at least 2-5 years of clinical practice including experience in leadership/management.

### Learning

The learning self-assessment reflects higher-level objective achievement in the cognitive, psychomotor, and affective domains at the end of the program (Table 2). The residents’ take-home messages highlighted their learning in relation to scholarship, including the importance of rigor and impact of academic work (n=7), critical thinking (n=2), clinical- academic work balance (n=2), career planning (n=2), and leadership (n=2). A number of residents also mentioned educational strategies (n=2) and approaches to educational innovation (n=1), self- directed learning (n=1) and networking (n=1).

**Table 2:** Université Laval’s Clinician Scholar Program impact on learning and behaviour (self-assessment)

		Pre-program Mean(SD)	Post-program Mean(SD)
**Learning**	**Knowledge (according to Bloom’s taxonomy**^12, 13^**)** (n=7) 0 – I have no knowledge of this concept 1 – I am able to **define** it 2 – I **understand** the principles associated with it 3 – I am able to **apply** this concept’s principles in my academic practice 4 – I am able to use this concept to **analyze** scholarly works 5 – I am able to **synthesize** the information relating to this concept and to teach it 6 – I am able to use this concept to **evaluate** my own work and the work of others		
	Conceptual frameworks in research, education, and management	0.0 (0.0)	4.3 (1.2)
	Scholarly project	1.0 (0.8)	4.3 (1.3)
	Curriculum development	0.4 (0.5)	4.3 (1.3)
	Information technologies in academic practice	1.6 (1.0)	4.4 (0.5)
	**Skills (according to Simpson’s taxonomy**^14^**)** (n=7) 0 – I have no understanding of this skill 1 – I am able to **identify** the situations where this skill is required 2 – I am **preparing** for practicing these skills 3 – I demonstrate this skill when guided (**close supervision**) 4 – I need assistance in complex situations or to validate my practice (**distant supervision**) 5 – I adapt my practice of this skill according to context (**independent practice**) 6 – I am **creating** new ways to practice this skill		
	Patient assessment through a learner case review	2.4 (1.3)	4.6 (0.5)
	Communication skills	3.9 (1.9)	4.7 (0.5)
	Teaching strategies	1.4 (1.0)	4.7 (0.8)
	Critical reading skills	2.7 (1.5)	4.4 (0.5)
	Personal management skills	3.3 (1.7)	5.0 (0.6)
	Interpersonal management skills/teamwork	3.6 (1.8)	4.9 (0.9)
	**Attitudes (according to Krathwohl’s taxonomy**^15^**)** (n=7) 0 – I am not entirely open to this approach 1 – I am **open** to this approach 2 – I am able to **contribute** to discussions on the topic 3 – I am able to **criticize** this approach 4 – I am able to **express my opinion** on this approach 5 – I am **demonstrating** the principles of this approach in my practice		
	Scholarship (working with rigour, getting peer assessment of one’s work, and disseminating it)	1.4 (1.6)	4.6 (0.8)
	Self-directed learning (identifying one’s training needs/objectives, implementing relevant strategies/resources, self-evaluation of learning)	2.4 (1.9)	4.6 (0.5)
	Critical thinking	1.7 (1.1)	4.4 (0.5)
	Ethics of academic practice	1.7 (1.5)	4.3 (1.0)
**Behaviours**	**Behaviours** (n=7) 0 – Non applicable 1 – I need **close supervision** 2 – I need **distant supervision**, i.e. occasional assistance to validate my actions or to get help in complex situations 3 – I am **independent**: I felt ready to practice on my own using this competency 4 – I feel like a **mentor** : I am able to teach this skill and provide guidance to others		
	Assessing and taking charge of complex clinical situations	2.0 (1.3)	2.7 (1.4)
	Discussing the importance of the three academic domains, i.e. research, education, and management, and the interconnection between them	1.2 (0.8)	3.2 (0.4)
	Defining a research question	1.3 (0.8)	2.8 (0.4)
	Reviewing the literature relevant to the topic	1.3 (1.0)	3.2 (0.8)
	Planning methodology	1.2 (1.0)	2.3 (0.5)
	Determining the schedule for a project	1.2 (0.8)	3.5 (0.5)
	Managing project implementation	1.0 (0.6)	3.2 (0.4)
	Building a database	1.3 (1.0)	2.8 (0.4)
	Analyzing project results	1.5 (1.0)	2.5 (0.5)
	Carrying out small-group teaching	1.3 (1.0)	3.2 (1.0)
	Lecturing	1.2 (0.8)	2.8 (0.8)
	Writing a scientific article	1.2 (0.8)	2.7 (0.8)
	Preparing and presenting a poster	1.0 (0.0)	3.2 (0.8)
	Collaborating with colleagues in research, education, AND leadership/management positions	1.2 (0.8)	2.8 (1.5)
	Supervising students/residents	0.8 (0.8)	2.8 (1.5)
	Personal management skills (clinical, academic, and personal activities)	2.3 (0.8)	3.3 (0.5)
	Exercising leadership skills	0.8 (0.4)	2.0 (1.2)
	Exercising modalities of influence in management (power, authority, leadership, politics)	0.7 (0.5)	1.5 (1.4)
	Implementing a reflective approach in relation to my academic practice (portfolio)	1.0 (0.0)	3.0 (0.9)
	Planning academic career	1.2 (0.4)	3.2 (0.8)
	Content analysis of behaviours reported in the reflective texts written by residents (n=6) at the end of the program, for the 3 academic missions: Research • Working in a scholarly manner (n=4) Education • Using a range of clinical teaching strategies (n=3) • Using a range of small group teaching strategies (n=3) • Communicating effectively (n=2) • Acting as a resource for colleagues in a clinical teaching context (n=1) • Identifying one’s limits in the context of teaching (n=2) Leadership/management • Demonstrating personal (n=3) and interpersonal (n=2) management skills • Developing collaboration and networking (n=1) • Pursuing an academic career (n=4) • Taking on academic responsibilities early in career (n=3)		

### Behaviour

The perceived entrustment level for various academic competencies progressed throughout the program. In addition, content analysis of the reflexive texts written by residents (n=6) at program completion highlights the development/improvement of a number of behaviours associated with practice in the three academic domains (Table 2): graduates value the scholarship approach, understand their teaching role for which they use a range of strategies, and are confident about taking on academic responsibilities making use of their management skills and with the help of the network they have developed during their training year. More particularly, residents taking the Medical Education elective demonstrated an improvement of their OSTE score by 14.4% compared to the control group residents (6.6%).

### Results

**Figure 2:**
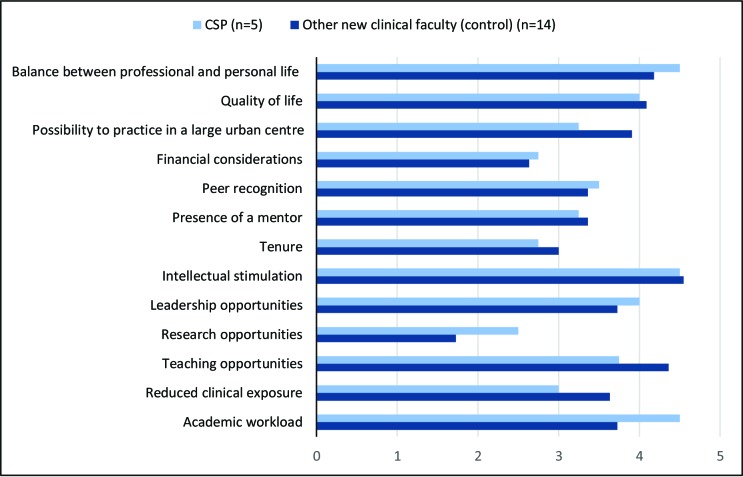
Factors influencing the intention to practice in an academic setting five years after graduation

Intention to practice in an academic setting five years of graduation was similar between graduates (4.8±0,45/5) and controls (4.9±0.36/5). Despite small sample size, available research/leadership opportunities, academic workload and seeking professional-personal life balance seem to influence CSP graduates’ intention to practice in an academic setting five years after graduation, whereas the possibility to practice in a large urban center, reduced clinical exposure and available teaching opportunities seem to have more influence on other new clinical faculty. (Figure 2 – Supplemental digital content).

CSP graduates’ academic productivity seemed relatively comparable to that of the control group. However, CSP graduates had slightly more opportunities for national outreach (Table 3 – Supplemental digital content). All CSP graduates joined an academic practice within five years of program completion (4 within and 3 outside the Laval University network).

## Discussion

The CSP at Université Laval offers a francophone training environment for a new wave of academic family physicians working towards a scholarship perspective. It is one of the rare programs targeting basic skills development in the entire range of academic medicine missions. Some schools have developed similar programs, but we found only two who have published their program description and/or evaluation. The first, from the Department of Family Medicine of the University of Western Ontario (London, ON, Canada) aimed “to produce academic family physicians who exhibit […] outstanding clinical skills, professional interest in the organization and transmission of knowledge, and a scholarly approach through research and skills of leadership.^19^ However, this program did not involve residents, but faculty members. Outcomes from this program included changes from private practice and lecturer to positions as assistant/associate/full professor and chairman/director, taking positions of responsibility for teaching and administering educational schemes. Graduates also produced substantial scholarly contributions. The other program we could find was the O’Connor Stanford Leaders in Education Residency program (Stanford University School of Medicine, California, USA) focuses mostly on teaching and scholarly projects, with leadership training components. This program increased confidence in teaching skills, and increased scholarly work output.^20^ While we noticed an interest in the CSP during residency program promotion events or information requests, more specific data about impact on applicants to our family medicine residency program would be helpful.

The reactions to the program are strongly positive, particularly for the academic medicine rotation. This is probably related not only with its content (principles of scholarship being new for many residents) and structure (project-based learning), but also by residents’ discovery of and involvement in a new community of academic practice. In general, our program resulted in learning, behaviours, and results that are comparable to those obtained in related programs, with similar challenges.^3^^,^^6^^,^^21^-25 Despite the absence of obvious impact of the CSP on the factors influencing intention for academic practice, it succeeds in providing tools supporting early academic career.

This program development and evaluation has certain limitations. To date, the CSP has mostly attracted applicants for the Medical Education track. The Clinical Research track might be more attractive if combined with a master’s degree, therefore facilitating a clinician-researcher career for graduates. Furthermore, the limited number of applicants for the Medical and Academic Leadership/Management track suggests that interest in and need for this type of training emerges later in one’s career. We are currently considering the possibility of offering this training after a few years of clinical practice in addition to the current third year residency enhanced-skills program. Other limitations result from political pressure to take on unattached patients in Quebec since 2014^26,^^27^ (with a growing number of family physicians focusing on clinical practice to the detriment of their academic involvement), which undeniably affected the program’s recruitment capacity and might explain decreased academic productivity following the program. Finally, the small size of the contingent of residents we can enroll and the choice we made of choosing an outcomes-based evaluation strategy do limit the conclusions we can draw from the program evaluation. Assessment of other aspects than outcomes (ex.: context, input, process^28^) would also be relevant for program directions. Nevertheless, our program evaluation strategy provided some qualitative data reinforcing that we meet the training needs of the new generation of family medicine faculty.

Université Laval’s CSP is a unique francophone residency program supporting new family physicians in an early academic career, balancing their clinical and academic roles with confidence in an environment fostering scholarship, mentorship, and networking. Program structure and content appears easily transferable to other specialties. We are confident that other medical schools should succeed in implementing similar programs in their own setting, to prepare the next generation of academic medical faculty. Key recommendations to implement similar programs (Box 1) include academic medicine core training, project-based learning with learner- centered objectives, relevant and authentic learning and assessment, and multi-level program evaluation approach. To meet other needs at a timely stage of career, programs should consider concomitant graduate studies and offering such training after a few years of clinical practice.

## Figures and Tables

Factors influencing the intention to maintain an academic practice five years after graduation assessed using a questionnaire based on three social cognitive theories: **theory of reasoned action**^29^, **theory of planned behaviour**^30^ and **interpersonal behaviour theory**^31^. CSP: clinician scholar program graduates.

## Footnotes

1 As stated in the 2014 edition of the *Tri-Council Policy Statement: Ethical Conduct for Research Involving Humans* (http://www.pre.ethics.gc.ca/eng/policy-politique/initiatives/tcps2-eptc2/chapter2-chapitre2/): “*Quality assurance and quality improvement studies, program evaluation activities, and performance reviews, or testing within normal educational requirements when used exclusively for assessment, management or improvement purposes, do not constitute research for the purposes of this Policy, and do not fall within the scope of REB review.”*

## Appendix A

Box 1 – Key recommendations for development, implementation and program assessment of an academic medicine program Based on the program evaluation results and consequent reflective analysis by the program committee members, further academic medicine programs should: Consider academic medicine core training at the program outset, to ensure strong bases for all residents, networking and engagement in their new community of practice. Focus on learner-centered objectives, using project-based learning to foster intrinsic motivation for learning. Engage residents in “real life”: help them manage clinical, academic and personal life schedules, using a horizontal curriculum; use relevant and authentic assessment strategies, having them build their teaching dossier as a portfolio documenting their competency achievement through the program. Encourage residents to pursue graduate studies concomitantly with their third-year clinical scholar residency program. Consider offering an academic leadership program after a few years of clinical practice, since interest in and need for training as an academic leader seems to emerge later in career. Adopt a multi-level program evaluation approach to foster scholarship in this field and provide evidence-based support for further program development. Assessment of other aspects than outcomes (such as context, input or process) would also be relevant for program directions.

## Appendix B

**Table 3:** Academic productivity among CSP graduates and other new clinical faculty who joined the Department of Family Medicine and Emergency Medicine between 2011 and 2015

Scholarship demonstrated by CSP graduates and other new clinical faculty		
	CSP (n=5)	New clinical faculty (control group) (n=14)
Teaching (%) • Classroom – pre-graduate • Classroom – post-graduate (residency) • Clinical training / supervision • Learner assessment (certification examinations) • Teaching innovations	60% 80% 80% 10% 80%	43% 86% 100% 7% 86%
Research (mean(SD)) • Number of projects as principal investigator • Number of projects as collaborator • Number of funded projects	2.1 (1.5) 0 0.4 (0.3)	0.1 (0.3) 0.2 (0.4) 0
Leadership & administration (%) • Academic • Clinical • Politics	60% 20% 0%	64% 57% 7%
Dissemination (mean(SD)) • Peer-reviewed journal publications (first author) • Communications (international) • Communications (national) - Papers (oral presentations) - Posters - Workshops	0.3 (0.4) 0 0 0.8 (0.8) 0.8 (1.1)	0 0 0 0.2 (0.4) 0.2 (0.3)

CSP : Clinician Scholar Program

## Appendix C

**Competencies associated with the CanMEDS scholar and leader/manager roles included in Université Laval’s Clinician Scholar Program (Quebec City, Canada)**

The competencies to be developed within the program were identified by a consensus of experts (program committee and teaching advisor) based on the College of Family Physicians of Canada (CFPC) accreditation standards for Clinician Scholar Programs, the literature on academic medicine competencies^a-e^, always embedded in the CanMEDs-family medicine framework^f^. The competencies related to clinical work with patients remained the same as those required under the family medicine residency program, since the CSP is aimed at maintaining acquired skills while developing greater independence for practice.

**Table:**

Program track			Competency	Entrustment levels (The numbers in parentheses indicate program expectations (in months) when each level must be achieved)		
Research	Education	Leadership		Close supervision	Distant supervision	Independent
			SCHOLAR			
x	x	x	Working from a scholarship perspective	Does not work from a scholarship perspective	Develops scholarly projects in a rigorous manner and requests from peers to review his or her work (8)	Submits his or her work for dissemination at the national or international level (13)
x	x	x	Building practice on recognized conceptual frameworks (theories, models, and best practices)	Builds research/education/ leadership strategies that include a limited number of conceptual frameworks	Builds research/education/ leadership strategies that include some conceptual frameworks that were suggested to him or her (3)	Builds research/education/ leadership strategies after having identified and analyzed the relevant conceptual frameworks (11)
x	x	x	Identifying the scientific literature that is relevant to his or her work using the appropriate databases	Needs close supervision from a mentor to conduct a literature search	Identifies the relevant literature using customary databases in his or her field (5)	Acts as a mentor to guide his or her colleagues in their literature search and directs them to more specialized sources when needed (11)
x			Providing constructive criticism of colleagues’ academic work	Comments mainly on the strengths and refers to a few points for improvement in a cursory manner	Provides balanced comments of strengths and points for improvement (8)	Provides constructive comments of strengths and points for improvement, supported by references, and offers relevant rectifications (13)
	x		Adopting a clinical “coaching” approach in his or her daily supervision activities*	Mostly validates the clinical conduct of the learner. Acts intuitively or understands and applies some recognized educational principles in relation to clinical supervision. Maintains at times a supervisor-trainee hierarchy.	Teaches various CanMEDS-FM competencies along with validating clinical conduct. Analyzes educational principles and identifies those that are most relevant to use depending on the clinical supervision situation, and encourages a “learning position” (11)	Acts as a leader** and scholar in relation to clinical supervision (14)
	x		Adopting a competency “coaching” approach throughout the learner’s training*	Acts intuitively or applies some recognized educational principles in relation to feedback and mentorship. Maintains at times a supervisor-trainee hierarchy.	Refers to a set of educational principles and identifies those that are most relevant to use depending on the feedback and/or mentorship situation, and encourages a “learning position” (11)	Acts as a leader** and scholar in relation to feedback and mentorship (14)
	x		Developing a training curriculum outside the clinical setting*	Acts intuitively when planning the curriculum.	Refers to a set of educational principles and applies those that are most relevant to his or her training activity outside the clinical setting (8)	Demonstrates leadership** and scholarship in curriculum development (including program assessment) (13)
x	x	x	Applying a range of **teaching strategies** in his or her training activities	Teaches intuitively and prefers lecturing to other educational strategies.	Includes some interactive strategies in his or her teaching (1)	Includes a variety of teaching strategies, encouraging collaborative learning in most training activities (3)
	x		Adapting the program to the needs of **learners with difficulties***	Acts intuitively or understands and applies some recognized educational principles to support learners with difficulties	Analyzes educational principles and identifies those that are most relevant to the learning plan of a learner with difficulties (11)	Demonstrates leadership** and scholarship to support learners with difficulties (14)
x	x	x	Documenting his or her learning process in a **portfolio**	Has an incomplete or poorly structured portfolio.	Documents his or her activity planning and academic production in a thorough and structured fashion (3)	Documents the acquired skills through feedback documents and reflective texts (8)
			LEADER			
x	x	x	Managing his or her time and priorities with a view to reconciling his or her **professional obligations (clinical and academic)** and **personal** life	Has difficulty prioritizing his or her professional obligations when faced with multiple requirements. Devotes too much time or not enough time to personal needs. Solves scheduling conflicts with difficulty or delay, or requires several supervisor interventions in order to ensure his or her practice’s effective management	Usually prioritizes appropriately his or her professional obligations when faced with multiple requirements. Is generally able to set adequate time aside for personal needs. Most of the time solves scheduling conflicts and rarely requires supervisor intervention in order to ensure his or her practice’s effective management (6)	Prioritizes consistently and appropriately his or her professional obligations when faced with multiple requirements. Consistently sets adequate time aside for personal needs. Solves scheduling conflicts consistently and effectively, and is managing his or her practice productively (12)
x	x	x	Using **information technologies** effectively to ensure his or her academic practice’s efficient functioning	Uses basic information technology functionalities (for example, word-processing and presentation software)	Uses appropriate advanced information technology functionalities in his or her academic practice (for example, table of contents, and reference and bibliography management software to cite sources) (3)	Uses advanced and/or interactive information technology functionalities to improve his or her academic practice (for example, bibliography management software for scientific articles database management, and interactive survey to make presentations more dynamic) (13)
x	x	x	Demonstrating **leadership** in the context of his or her scholarly project	Requires close assistance in order to assume the administrative roles related to his or her project. Needs stimulation in order to participate actively in working meetings	Is sometimes slow to assume the administrative tasks related to his or her project. Participates actively in working meetings (5)	Anticipates and assumes the administrative tasks related to his or her project in a timely manner. Demonstrates leadership in the context of working meetings (11)
		x	Implementing **change** in his or her setting	Requires close supervision to implement changes in his or her practice, requiring assistance for specialized aspects. Reacts to challenges/difficulties.	Needs distant supervision to implement changes in his or her practice and applies recognized theoretical principles in management. Anticipates challenges/difficulties. (11)	Acts as a mentor with his or her peers and supports them in the implementation of change in their practice. Evaluates his or her interventions on the basis of recognized theoretical principles in management (13)
		x	Experimenting with a range of **leadership **styles in order to advance his or her projects	Exercises leadership spontaneously without knowledge of leadership styles	Consciously experiments a number of leadership styles in his or her interventions (8)	Adjusts his or her leadership style to the situation (11)
		x	Exercising a range of **management skills**	Exercises some management skills in a spontaneous and intuitive manner	Exercises a number of management skills in a conscious manner (11)	Acts as a mentor with his or her peers when exercising management skills (14)
x	x	x	**Soliciting** the relevant university and healthcare system authorities for the purpose of his or her projects	Requires close supervision from some relevant authorities for the purpose of his or her projects.	Needs distant supervision from a number of relevant authorities for the purpose of his or her projects. (5)	Acts as a mentor with his or her peers to help them soliciting relevant authorities for the purpose of their projects (14)
x	x	x	Exercising various **modalities of influence** in relation to management (power, authority, leadership, political skill)	Exercises some modalities of influence in his or her management activities in a spontaneous and intuitive manner	Consciously experiments with a number of modalities of influence in his or her management activities (8)	Adjusts his or her modalities of influence to the management activity concerned (11)

* Fundamental teaching activities framework^5^. In order to meet the entrustment level requirements for clinician scholars in the early stages of their career, residents must fulfill all requirements of the “Consistently applies fundamental and advanced educational tasks” level of the CFPC standards.

** Leadership means that the teacher is considered as a resource person or a mentor by his or her peers.
